# Erythrocyte glutathione transferase: a general probe for chemical contaminations in mammals

**DOI:** 10.1038/cddiscovery.2016.29

**Published:** 2016-05-23

**Authors:** A Bocedi, R Fabrini, O Lai, L Alfieri, C Roncoroni, A Noce, JZ Pedersen, G Ricci

**Affiliations:** 1 Dipartimento di Scienze e Tecnologie Chimiche, Università degli Studi di Roma ‘Tor Vergata’, Rome, Italy; 2 Direzione Operativa Produzioni Zootecniche, Istituto Zooprofilattico Sperimentale del Lazio e della Toscana ‘M. Aleandri’, Rome, Italy; 3 Unità di Nefrologia e Ipertensione, Dipartimento di Medicina dei Sistemi, Università degli Studi di Roma ‘Tor Vergata’, Rome, Italy; 4 Dipartimento di Biologia, Università degli Studi di Roma ‘Tor Vergata’, Rome, Italy

## Abstract

Glutathione transferases (GSTs) are enzymes devoted to the protection of cells against many different toxins. In erythrocytes, the isoenzyme (e-GST) mainly present is GSTP1-1, which is overexpressed in humans in case of increased blood toxicity, as it occurs in nephrophatic patients or in healthy subjects living in polluted areas. The present study explores the possibility that e-GST may be used as an innovative and highly sensitive biomarker of blood toxicity also for other mammals. All distinct e-GSTs from humans, *Bos taurus* (cow), *Sus scrofa* (pig), *Capra hircus* (goat), *Equus caballus* (horse), *Equus asinus* (donkey) and *Ovis aries* (sheep), show very similar amino acid sequences, identical kinetics and stability properties. Reference values for e-GST in all these mammals reared in controlled farms span from 3.5±0.2 U/g_Hb_ in the pig to 17.0±0.9 U/g_Hb_ in goat; such activity levels can easily be determined with high precision using only a few microliters of whole blood and a simple spectrophotometric assay. Possibly disturbing factors have been examined to avoid artifact determinations. This study provides the basis for future screening studies to verify if animals have been exposed to toxicologic insults. Preliminary data on cows reared in polluted areas show increased expression of e-GST, which parallels the results found for humans.

Glutathione transferases (GSTs) are a superfamily of detoxifying enzymes devoted to inactivate a large variety of different toxic compounds.^1,2^ They catalyze the conjugation of glutathione to many organic compounds, so that it can be more easily eliminated from the organism.^1,2^ Moreover, they can act like ligandins sequestering toxic molecules including iron nitric oxide complexes.^3^ In mammalian species, the dimeric cytosolic GSTs are abundantly expressed in many tissues and grouped into seven distinct isoenzyme classes termed alpha, pi, mu, omega, sigma, theta and zeta.^1,2^ The only GST belonging to the pi class is GSTP1-1, an interesting enzyme that is mainly present in erythrocytes, brain, lung and skin. This isoenzyme is also involved in the modulation of the apoptotic cascade through its interaction with cJNK.^4^ Recently, it has been observed that the human erythrocyte GSTP1-1 (e-GST) is overexpressed in the case of increased blood toxicity as it occurs in healthy subjects living in polluted areas and in nephrologic patients under conservative or dialytic therapies.^5–8^ Interestingly, the expression of this enzyme does not fulfill an instantaneous snapshot of the blood toxicity, but an average value over a time span of about 2/3 months (corresponding to the mean life of the erythrocyte) as it is exclusively expressed during erythropoiesis.^5^ Therefore, e-GST has been proposed as an innovative biomarker in man that is able to signalize a long-term exposition to environmental pollution, or to reveal the efficiency either of kidney function or of artificial dialytic techniques.^5–8^ A realistic hypothesis is that this specific enzyme could behave similarly also in other mammalian species. This study for the first time makes a comparison of the molecular and kinetic properties of e-GSTs from seven different mammal species. The presence of interfering factors like the occurrence of the inactive oxidized form of e-GST^9^ has also been examined.

## Results

### Molecular properties of e-GSTs from different mammalian species

The amino acid sequences of e-GSTs from swine, goat, cow, sheep and two equine species display extraordinary similarity among them, and also with the human enzyme. Most of these GSTs show >85% of sequence identity with the human isoform (Supplementary Figure S1 and Supplementary Table S1). Of interest is the strict conservation of the four cysteines (Cys14, Cys47, Cys101 and Cys169) that confer peculiar redox sensitivity to this enzyme. Both in human and horse e-GSTs, many oxidizing chemicals may induce the formation of an intra-chain disulfide involving the two highly reactive cysteines, that is, Cys47 and Cys101.^9,10^ These oxidized forms are completely inactive, but they can be reactivated under reducing treatment with DTT at alkaline pH values. The presence of a detectable inactive oxidized form of GSTP1-1 has been recently found in salivary samples of healthy human subjects.^11^ The possible presence of such oxidized forms in the blood of different mammals will be explored below.

### Kinetics and stability properties of e-GSTs from different mammalian species

The similarity of the primary structures of these distinct e-GSTs suggests almost identical kinetics properties. As expected, *K*_m_ values for GSH and CDNB are very similar (Table 1) and *k*_cat_ values also range between 75–85 /s for all mammalian species selected for this study (Table 1). Thus, all these mammalian isoforms of e-GST appear kinetically very similar.

Essential for a possible use of these enzymes in screening analysis is their stability upon storage. In intact erythrocytes, almost all these transferases display very high stability for many days when stored at 4 °C (no statistically significant decrease in activity after 7 days). The exception is the pig e-GST that is stable only for 2 days, displaying a loss of 25% of its original activity after 7 days (Supplementary Table S2). Storage of blood samples at −20 °C must be avoided as it causes partial inactivation of e-GSTs from all species (data not shown).

### Activity of e-GST in bovine erythrocytes

**Figure 1:**
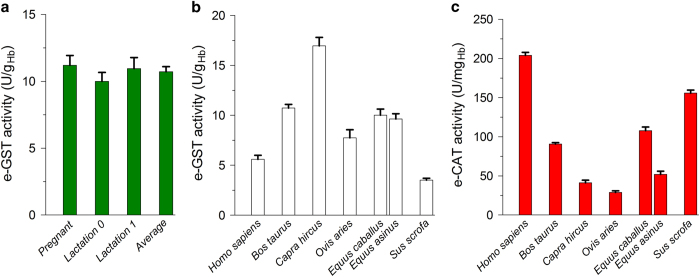
e-GST and e-CAT in selected mammalian species. (**a**) Bovine e-GST in different physiological conditions. Values are the mean of three sets of measurements performed on 40 cows during pregnancy, during lactating phase at 1 month (lactation 0) and at 4 months postpartum (lactation 1). (**b**) e-GST in humans and in selected mammalian species. (**c**) e-CAT in humans and in selected mammalian species. Error bars are the S.E.M.

e-GST activity has been measured in 40 pregnant cattle reared in controlled farms. Pregnant cattle display 11.2±0.7 U/g_Hb_, a value higher than that found in humans (5.8±0.4 U/g_Hb_) (Figures 1a and b). For these animals, we tested if the e-GST level could be influenced by different physiological conditions, that is, pregnancy, and during lactating phase, 1 month (10.0±0.7 U/g_Hb_) and 4 months (11.0±0.8 U/g_Hb_) after partum. In Supplementary Table S3 is reported the statistical significance among e-GST activities from the three different physiological conditions.

### e-GSTs in other mammalian species

The amount of e-GST in the animals tested appears species-specific (Figure 1b). Goat shows the highest level (17.0±0.9 U/g_Hb_), whereas pig expresses a relatively low amount of 3.5±0.2 U/g_Hb_. The activity of e-GST has been compared with the level of e-CAT, an enzyme often considered a biosensor of oxidative stress (Figure 1c). Notably, the concentration of e-CAT in humans was higher than in other mammalian species, exceeding five to six times the levels in goat and sheep. In Supplementary Tables S4 and S5 are reported the statistically significant differences obtained by comparing the levels of e-GST and e-CAT enzymatic activities in different species.

### Oxidized e-GST in bovine erythrocyte: reality or artifact?

**Figure 2:**
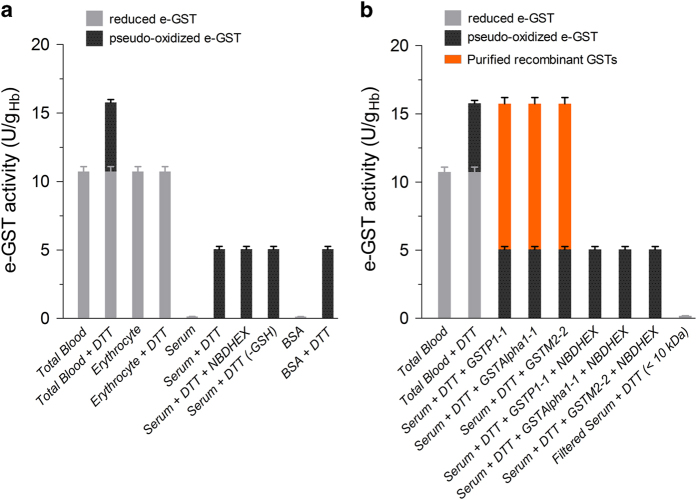
e-GST and pseudo-oxidized e-GST in bovine blood. (**a**) Mean level of e-GST in: total bovine blood, blood treated with the reducing agent DTT, isolated erythrocytes, isolated erythrocytes treated with DTT, bovine serum, bovine serum treated with DTT, bovine serum treated with DTT and incubated with the specific GST inhibitor NBDHEX (0.1 mM final concentration), and serum treated with DTT and assayed without the substrate GSH. The last two columns on the right report the reaction of purified BSA (in the same assay concentration of serum samples, 2.3 *μ*M) with CDNB (1 mM, pH 6.5) before and after reduction with DTT. (**b**) Recombinant purified GSTP1-1, GSTA1-1 and GSTM2-2 were added to bovine serum after DTT reduction to reach the reported activities. Each samples were then incubated with the specific GST inhibitor NBDHEX (0.1 mM final concentration). Last column on the right shows the activity in serum treated with DTT and filtered by Ultracel cutoff 10 kDa (Amicon, Merck Millipore, Darmstadt, Germany). Error bars are the S.E.M.

As mentioned above, the human GSTP1-1 may form an oxidized enzyme involving the two more reactive cysteines (i.e., Cys47 and Cys101).^9^ The resulting oxidized enzyme is fully inactive.^9^ The presence of oxidized GSTP1-1 has recently been discovered in human saliva,^11^ but it has never been observed in mammalian blood. After incubation of hemolyzed total blood with DTT, we observed a conjugation rate of GSH with CDNB higher than that found in the absence of reducing treatment, suggesting the presence of a significant, not spurious amount of oxidized e-GST (Figure 2a). Surprisingly, this additional activity was not recovered in isolated erythrocytes, but only in serum (Figure 2a). In a recent reinvestigation of the possible presence of GSTP1-1 in human serum, we concluded that it was absent or below the detection limit of the usual spectrophotometric assay,^12^ but we did not verify the possible occurrence of oxidized GSTP1-1. So far, no data are available in the literature concerning the presence of oxidized GSTP1-1 in bovine blood. Further experiments to clarify this point gave surprising results. In fact, the addition of NBDHEX, a strong and specific inhibitor of e-GST and other GST isoenzymes belonging to the alpha, pi and mu classes,^13^ did not suppress this additional GST activity (Figure 2b), and curiously the activity was also found in the absence of GSH (Figure 2a). Thus, the additional activity observed after reduction with DTT cannot be explained by the presence of oxidized GST in the samples.

### Reduced serum albumin simulates a pseudo-GST activity

The above findings indicate that an unknown sulfhydryl compound present in blood and formed during DTT treatment is able to react with CDNB at a high rate. Low molecular mass disulfides can be excluded, because serum depleted of high mass components by filtration does not show any reactivity with CDNB (Figure 2b). Thus, our attention instead focused on proteins and BSA was a likely candidate. This protein is highly concentrated in serum and displays 17 disulfides that can be reduced under DTT treatment. Purified BSA, reduced as described under Materials and Methods section, used at same serum concentration reacts with CDNB with a velocity identical to that observed using the bovine serum (Figure 2a). This suggests that reduced BSA is responsible for the apparent extra GST activity detected in our samples.

### GSTP1-1 in intact erythrocytes is resistant to oxidative insults

**Figure 3:**
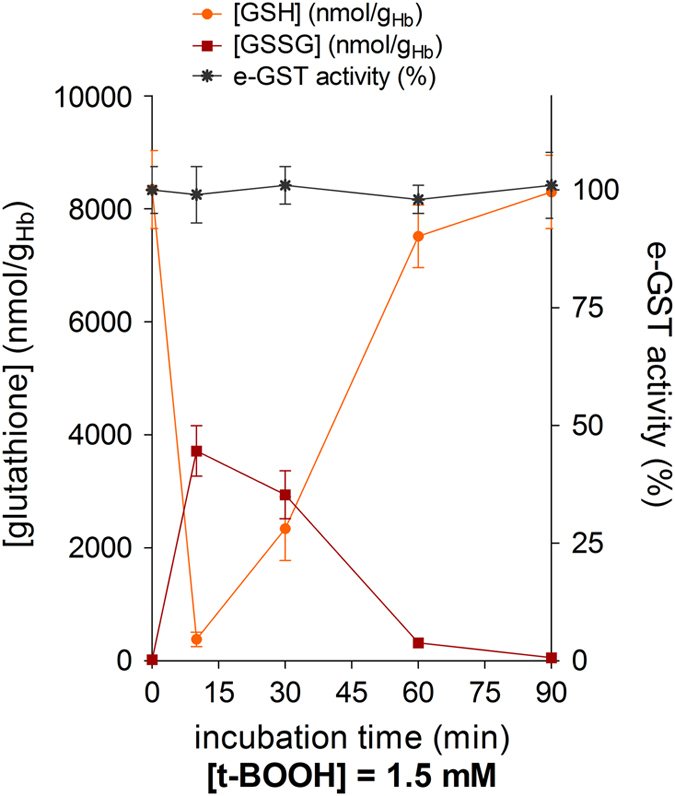
Changes of GSH, GSSG and e-GST during incubation of bovine blood with peroxide. Bovine blood (1 ml) was incubated with 1.5 mM t-BOOH, and at various times, GSH, GSSG and e-GST were measured. Experimental points are the mean of three different determinations. Error bars are the S.E.M.

The absence of oxidized e-GST in normal bovine blood samples does not exclude its presence after severe oxidative stress. Thus, the levels of GSH, GSSG and GSP1-1 were determined after incubation of intact erythrocytes with t-BOOH, a well-known trigger of oxidative stress. Although GSH is rapidly oxidized to GSSG, the activity of e-GST remained unchanged, indicating a strong resistance to oxidants (Figure 3). In addition, the enzymatic cell protection system (glutathione reductase) is rapidly involved and causes a restoration of the original concentration of GSH in <2 h.

### e-GST levels in cows raised in highly polluted areas

Preliminary data obtained analyzing blood samples from 10 cows reared in farms located in a well-known polluted area (River Sacco valley, located in the Frosinone district, Lazio, Italy) showed that e-GST is overexpressed, displaying an average value of 18±3 U/g_Hb_, much higher than that found in the samples obtained from controlled farms located in unpolluted areas (10.7±0.4 U/g_Hb_) (Figure 1a). This behavior closely parallels the one found for humans living in the same area.^7^

## Discussion

This study shows a first comparison of kinetics and molecular properties of different e-GSTs from mammalian species. An examination of sequence data and kinetics properties of the e-GSTs from pig, goat, cow, sheep and horse demonstrated that all these enzymes are very similar and almost identical to the human GSTP1-1 (Table 1). It is interesting that the basic level of e-GST activity does not seem to be connected with the general level of oxidative stress in the animal; in fact a comparison between e-GST and e-CAT activities shows completely different species profiles (Figure 1).

All these e-GSTs display an extraordinary stability at 4 °C except for the pig e-GST that began to lose activity after 2 days (Supplementary Table S2). As expected, the expression of e-GST activity is species-specific; the lowest levels were found in humans and in the pig, whereas the highest activity was observed in the goat. Physiological phases like lactation or pregnancy gave no statistically significant differences (Figure 1a) (Supplementary Table S3). The present study also explores the possibility that the oxidized inactive form of e-GST may be present in blood, given that two critical cysteines, Cys47 and Cys101 (the latter corresponding to Cys103 in pig e-GST) are strictly conserved in all mammals tested, and that they form a disulfide both *in vivo* and *in vitro* as proved for the human enzyme.^9,11^ Our data indicate that oxidized e-GST, if present, is below the detection limit of the assay. Even strong oxidative stress does not produce appreciable amounts of oxidized e-GST. An artifact GST-like activity is observed in serum after DTT reduction, mainly due to hyper-reactivity of a few cysteines of serum albumin. This phenomenon must be seriously considered for a correct determination of e-GST in blood.

Preliminary results on cows reared in farms residing in a highly polluted area confirm that the e-GST activity is a highly sensitive parameter for detecting increased toxicity levels. As observed in humans,^7^ the overexpression of e-GST in animals is likely a defense response to an increased blood toxicity, and this behavior resembles the increased production of white blood cells in case of bacterial infections. An increased e-GST level in animals is therefore an alarm signal that must be followed up by more accurate investigation to assess the chemical nature of the contaminants.

All distinct e-GSTs from humans, *Bos taurus* (cow), *Sus scrofa* (pig), *Capra hircus* (goat), *Equus caballus* (horse), *Equus asinus* (donkey) and *Ovis aries* (sheep) show very similar amino acid sequences, identical kinetics and stability properties. Reference values for e-GST activity in all these mammals have been determined. Possibly disturbing factors have been examined to avoid artifact determinations. Preliminary data on cows reared in polluted areas show increased expression of e-GST that parallels the results found for humans. This study provides the basis for future screening studies to verify if animals have been exposed to toxicologic insults.

## Materials and Methods

### Ethics statements

70 healthy volunteers were recruited for blood recovery after written consent. The study conformed to the standards set by the Declaration of Helsinki. The study was approved by local Ethics Committee of University of Rome Tor Vergata.

Animal blood samples have been recovered according to EU Directive 2010/63/EU for animal experiments. The study was carried out in five private dairy farms located on private land, with permission from the owners. This study has been performed employing the blood samples collected as part of farm routine health controls. Specific permissions from Italian Ministry of Health (Ordinance n. 153/2001-A) for animal care and use, and from the Ethics Committee of the University of Rome Tor Vergata, were obtained for these locations/activities and study. As the study has been carried out on dairy cattle and farm animals, the field study did not involve endangered or protected species. None of the animals were sacrificed for this study.

### Blood samples

All animals tested were randomly chosen from controlled farms of the Latium and Tuscany countrysides. We examined in particular: *Bos taurus* (*n*=40), *Sus scrofa* (*n*=18), *Capra hircus* (*n*=20), *Equus caballus* (*n*=14), *Equus asinus* (*n*=14) and *Ovis aries* (*n*=15). For the *Bos taurus* species, 40 animals were tested during pregnancy and during lactation, after 1 month and after 4 months postpartum, respectively. As no relevant changes in e-GST levels were observed for bovine species (see Results section), all other mammalian species were tested without considering these physiological phases. Peripheral blood samples were collected from the jugular vein in tubes with tripotassium EDTA in adult subjects. The samples were collected by a veterinarian using the proper procedure of blood collection, and stored at 4 °C for no more than 2 days. Furthermore, a complete blood cell count (hemogram) was performed within 24 h using an automated counter Cell-Dyn 3700, 12 parameters (Abbott Laboratories, Abbott Park, IL, USA) to assess animal health status, mainly the hemoglobin value.

For human blood, 70 healthy volunteers with normal renal function and no history of diabetes mellitus served as healthy controls. Blood samples were collected from the antecubital vein and stored into tripotassium EDTA tubes at 4 °C.^5^

### Materials

Reduced glutathione (GSH), oxidized glutathione (GSSG), 1-chloro-2,4-dinitrobenzene (CDNB), dithiotreitol (DTT), chemicals for Lowry solutions, *S*-hexylglutathione sepharose 6B, *tert*-butyl hydroperoxide (t-BOOH), EDTA, *N*-ethylmaleimide, 4-chloro-7-nitrobenzofurazan, 6-mercapto-1-hexanol, 3-bromopyruvate, bovine serum albumin (BSA) and all other reagents were purchased from Sigma-Aldrich (St. Louis, MO, USA). The compound 6-(NBD-4-ylthio-)hexanol (NBDHEX) was synthesized as described previously.^13^ Human e-GST was expressed in *E.coli* and purified as described,^14^ whereas GSTA1-1 and GSTM2-2 were expressed and purified according to a previous study.^15^

### e-GST activity

e-GST activity was determined with a spectrophotometric assay at 340 nm (37 °C). 40 *μ*l of whole blood were diluted in 1 ml of bi-distilled water causing erythrocyte hemolysis. After 2 min, 0.1 ml samples were diluted to a final volume of 1 ml containing 1 mM GSH, 1 mM CDNB in 0.1 M potassium phosphate buffer, pH 6.5, according to the standard procedure of Habig *et al*.^16^ Results were expressed as enzyme units per gram of Hb (U/g_Hb_);^5^ 1 unit represents the amount of enzyme that catalyzes the conjugation of 1 micromole of GSH to CDNB in 1 min at 37 °C. e-GST activity in isolated erythrocyte was determined as above after hemolysis of collected erythrocytes contained in 40 *μ*L of total blood.

### Kinetics parameters

The *K*_m_ value for GSH was obtained by reacting about 0.5 *μ*g of purified mammalian e-GSTs with variable amounts of GSH (from 0.02  to 2 mM) in the presence of 1  mM CDNB, in 0.1 M potassium phosphate buffer, pH 6.5 (25 °C). The *K*_m_ value for CDNB was obtained by reacting 0.5 *μ*g of purified e-GSTs of mammals with variable amounts of CDNB (from 0.05  to 2 mM) in the presence of 1 mM GSH, in 0.1 M potassium phosphate buffer, pH 6.5 (25 °C). From these experimental data, *k*_cat_ values (at saturating CDNB and GSH) were also calculated.

### Erythrocyte catalase activity

Erythrocyte catalase (e-CAT) activity was determined with a spectrophotometric assay at 240 nm (25 °C). 5 *μ*l of hemolyzed blood was diluted in 1 ml of potassium phosphate buffer (0.05 M, pH 7.0) with EDTA 0.1 mM, and finally in 10 *μ*l of H_2_O_2_ (1 M) according to the standard procedure of Beers and Sizer.^17^ Results were expressed as enzyme units per gram of Hb (U/g_Hb_): 1 unit represents the amount of enzyme that catalyzes the decomposition of 1 micromole of H_2_O_2_ in 1 min at 25 °C.

### Reactivation procedure for oxidized e-GST

Different hemolyzed blood samples (total blood, isolated erythrocytes or serum) and purified BSA approximately at the same concentration present in hemolyzed blood (23 *μ*M), were all incubated for 60 min at 37 °C in a dry block thermostat with 1 mM final concentration of DTT and 0.01 M potassium phosphate buffer (pH 8.0).

### Erythrocyte GSH and GSSG determination

Whole blood (0.5 ml) were reacted with 0.05 ml of 310 mM *N*-ethylmaleimide, and then GSH and GSSG were determined by following the procedure described previously.^18^

### Sequence analysis

Amino acid sequences were selected from NCBI and UniProtKB databases: *Homo sapiens* (code: P09211), *Bos taurus* (code: NP_803482.1), *Capra hircus* (code: Q9TTY8.2), *Equus caballus* (code: XP_001498156.1), *Ovis aries* (code: XP_004019771.1) and *Sus scrofa* (code: M3V836). Sequence alignment and percentage of similarities were derived using ClustalW v2.1^19^ available on the website of EMBL.

### Purification of e-GST

Purification of e-GST from hemolyzed erythrocytes of all species tested, was performed with a single-step affinity chromatography method using *S*-hexylglutathione sepharose 6B.^20^ Protein concentration was determined using the procedure described by Lowry *et al*.^21^

### Statistical analysis

All data were expressed as mean±S.E.M. *P*-values reported in the text and in tables have been estimated on the basis of the mean±S.D. All continuous variables were checked for normality using the Levene’s test. One-way analysis of variance (ANOVA) was employed to compare the data between different species. To compare the e-GST activity values of *Bos taurus,* in different physiological conditions, One-way analysis of variance (ANOVA) was used. A value of *P*<0.05 was considered statistically significant. Data were processed using the statistical software MedCalc (Mariakerke, Belgium).

## Figures and Tables

**Table 1:** A comparison of the kinetics parameters of mammalian e-GSTs^a^

	*K_*m*_*		*k_cat_* (/*s*)
	*GSH (mM)*	*CDNB (mM)*	
*Homo sapiens*	0.11±0.01	1.0±0.1	79±5
*Bos taurus*	0.12±0.02	0.8±0.2	83±7
*Capra hircus*	0.14±0.02	0.9±0.1	85±6
*Ovis aries*	0.10±0.01	0.8±0.2	77±8
*Equus caballus*	0.10±0.02	0.8±0.2	82±6
*Sus scrofa*	0.10±0.02	0.9±0.1	75±7

a Data shown are the mean±S.E.M. from three distinct experiments.

## Abbreviations

DTT: dithiotreitol
GSH: glutathione
GSSG: oxidized glutathione
GST: glutathione transferase
e-CAT: erythrocyte catalase
e-GST: erythrocyte glutathione transferase.
