# Supplementary material for: Erythrocyte glutathione transferase: a general probe for chemical contaminations in mammals
Source: Cell Death Discov. 2016 May 23;2:16029–. doi: 10.1038/cddiscovery.2016.29 (PMC4979413; doi:10.1038/cddiscovery.2016.29)
Supplement: Supplemental Information [file cddiscovery201629-s1.doc]

**Supplementary material**

**Supplementary Table 1.** Percent Matrix of Sequence Identity for mammalian e-GSTs

|  | *Bos*  *taurus* | *Capra*  *hircus* | *Ovis*  *aries* | *Equus*  *caballus* | *Sus*  *scrofa* |
| --- | --- | --- | --- | --- | --- |
| *Homo sapiens* | 86.19 | 84.29 | 86.21 | 89.05 | 83.65 |
| *Bos taurus* |  | 97.14 | 96.06 | 91.43 | 87.02 |
| *Capra hircus* |  |  | 96.06 | 90.48 | 86.54 |
| *Ovis aries* |  |  |  | 92.12 | 88.06 |
| *Equus caballus* |  |  |  |  | 89.42 |

**Supplementary Table 2**. Stability of e-GST activity in blood samples stored at 4 °C

|  | e-GST activity (%)  (2 days storage at 4 °C) | e-GST activity (%)  (4 days storage at 4 °C) | e-GST activity (%)  (7 days storage at 4 °C) |
| --- | --- | --- | --- |
| *Homo sapiens* | 99 ± 2 | 101 ± 4 | 100 ± 3 |
| *Bos taurus* | 101 ± 3 | 102 ± 4 | 102 ± 6 |
| *Capra hircus* | 101 ± 2 | 100 ± 1 | 102 ± 7 |
| *Ovis aries* | 103 ± 7 | 98 ± 9 | 98 ± 8 |
| *Equus caballus* | 102 ± 1 | 100 ± 7 | 102 ± 5 |
| *Equus asinus* | 101 ± 1 | 101 ± 6 | 101 ± 5 |
| *Sus scrofa* | 103 ± 3 | 102 ± 7 | 75 ± 8 |

**Supplementary Table 3.** Statistical significance among e-GST activities from three different physiological conditions of *Bos taurus**

|  | Lactation 0 | Lactation 1 |
| --- | --- | --- |
| Pregnant | n.s. | n.s. |
| Lactation 0 |  | n.s. |

*e-GST activities from Fig. 1A

**Supplementary Table 4.** Statistical significance among e-GST activities from different mammalian species*

|  | *Capra hircus* | *Equus caballus* | *Equus asinus* | *Sus scrofa* | *Ovis aries* |
| --- | --- | --- | --- | --- | --- |
| *Bos taurus* | *P* < 0.001 | n.s. | n.s. | *P* < 0.001 | n.s. |
| *Capra hircus* |  | *P* < 0.001 | *P* < 0.001 | *P* < 0.001 | *P* < 0.001 |
| *Equus caballus* |  |  | n.s. | *P* < 0.001 | n.s. |
| *Equus asinus* |  |  |  | *P* < 0.001 | n.s. |
| *Sus scrofa* |  |  |  |  | *P* < 0.001 |

*e-GST activities from Fig. 1B

**Supplementary Table 5.** Statistical significance among e-CAT activities from different mammalian species*

|  | *Capra hircus* | *Equus caballus* | *Equus asinus* | *Sus scrofa* | *Ovis aries* |
| --- | --- | --- | --- | --- | --- |
| *Bos taurus* | *P* < 0.001 | n.s. | *P* < 0.001 | *P* < 0.001 | *P* < 0.001 |
| *Capra hircus* |  | *P* < 0.001 | n.s. | *P* < 0.001 | n.s. |
| *Equus caballus* |  |  | *P* < 0.001 | *P* < 0.001 | *P* < 0.001 |
| *Equus asinus* |  |  |  | *P* < 0.001 | *P* < 0.001 |
| *Sus scrofa* |  |  |  |  | *P* < 0.001 |

*e-CAT activities from Fig. 1C

**Supplementary Figure 1**

*Homo sapiens* MPPYTVVYFPVRGR**C**AALRMLLADQGQSWKEEVVTVETWQEGSLKAS**C**LYGQLPKFQDGD 60

*Bos taurus* MPPYTIVYFPVQGR**C**EAMRMLLADQGQSWKEEVVAMQSWLQGPLKAS**C**LYGQLPKFQDGD 60

*Capra hircus* MASYTIVYFPVQGR**C**EAMRMLLADQDQSWKEEVVAMQSWLQGPLKAS**C**LYGQLPKFQDGD 60

*Equus caballus* MPPYTIVYFSVRGR**C**EAMRMLLADQGQSWKEEVVTVDTWMQGPLKAS**C**LYGQLPKFQDGD 60

*Ovis aries* MPPYTIVYFPTRGR**C**EAMRMLLADQDQSWKEEVVAMQSWLQGPLKAS**C**LYGQLPKFQDGD 60

*Sus scrofa* MPPYTITYFPVRGR**C**EAMRMLLADQDQSWKEEVVTMETWP--PLKPS**C**LFRQLPKFQDGD 58

*..**:.**..:*** *:*******.********::::* .**.***: *********

*Homo sapiens* LTLYQSNTILRHLGRTLGLYGKDQQEAALVDMVNDGVEDLR**C**KYISLIYTNYEAGKDDYV 120

*Bos taurus* LTLYQSNAILRHLGRTLGLYGKDQQEAALVDMVNDGVEDLR**C**KYVSLIYTNYEAGKEDYV 120

*Capra hircus* LTLYQSNAILRHLGRTLGLYGKDQREAALVDMVNDGVEDLR**C**KYVSLIYTNYQAGKEDYV 120

*Equus caballus* LTLYQSNAILRHLGRSLGLYGKDQREAALVDMVNDGVEDLR**C**KYVTLIYTNYEAGKEDYV 120

*Ovis aries* LTLYQSNAILRHLGRTLGLYGKDQREAALVDMVNDGVEDLR**C**KYVSLIYTNYEAGKEDYV 120

*Sus scrofa* LTLYQSNAILRHLGRSFGLYGKDQKEAALVDMVNDGVEDLR**C**KYATLIYTNYEAGKEKYV 118

*******:*******::*******:******************* :******:***: **

*Homo sapiens* KALPGQLKPFETLLSQNQGGKTFIVGDQISFADYNLLDLLLIHEVLAPG**C**LDAFPLLSAY 180

*Bos taurus* KALPQHLKPFETLLSQNKGGQAFIVGDQISFADYNLLDLLRIHQVLAPS**C**LDSFPLLSAY 180

*Capra hircus* KALPQHLKPFETLLSQNKGGQAFIVGDQISFADYNLLDLLRIHQVLAPS**C**LDSFPLLSAY 180

*Equus caballus* KALPGHLKPFETLLSQNQGGQAFIVGNQISFADYNLLDLLLIHQVLAPS**C**LDSFPLLSAY 180

*Ovis aries* KALPQHLKPFETLLSQNKGGQAFIVGDQISFADYNLLDLLRIHQVLAPS**C**LDSFPLLSAY 180

*Sus scrofa* KELPEHLKPFETLLSQNQGGQAFVVGSQISFADYNLLDLLRIHQVLNPS**C**LDAFPLLSAY 178

* ** :***********:**::*:**.************* **:** *.***:*******

*Homo sapiens* VGRLSARPKLKAFLASPEYVNLPINGNGKQ 210

*Bos taurus* VARLNSRPKLKAFLASPEHMNRPINGNGKQ 210

*Capra hircus* VARLNSRPKLKAFLASPEHVNRPINGNGKQ 210

*Equus caballus* VARLSARPKLKAFLASPEHVNLPINGNGKQ 210

*Ovis aries* VARLSARPKLKX-------VNRPINGNGKQ 203

*Sus scrofa* VARLSARPKIKAFLASPEHVNRPINGNGKQ 208

*.**.:***:* :* ********

**Supplementary Figure 1.** **GSTP1-1 (e-GST) sequences alignment in selected mammalian species*.*** Data obtained from NCBI and UniProtKB databases. Cysteines are colored in yellow, Asterisks (*) indicate the residues fully conserved in all mammalian sequences. Colon (:) indicate conservative mutations. Point (.) indicates semi-conservative mutations.
